# Comparative Transcriptome Profiling of Chilling Stress Responsiveness in Two Contrasting Rice Genotypes

**DOI:** 10.1371/journal.pone.0043274

**Published:** 2012-08-17

**Authors:** Ting Zhang, Xiuqin Zhao, Wensheng Wang, Yajiao Pan, Liyu Huang, Xiaoyue Liu, Ying Zong, Linghua Zhu, Daichang Yang, Binying Fu

**Affiliations:** 1 Engineering Research Center for Plant Biotechnology and Germplasm Utilization, Ministry of Education, State Key Laboratory of Hybrid Rice, College of Life Sciences, Wuhan University, Wuhan, China; 2 Institute of Crop Sciences/National Key Facility for Crop Gene Resources and Genetic Improvement, Chinese Academy of Agricultural Sciences, Beijing, China; Michigan State University, United States of America

## Abstract

Rice is sensitive to chilling stress, especially at the seedling stage. To elucidate the molecular genetic mechanisms of chilling tolerance in rice, comprehensive gene expressions of two rice genotypes (chilling-tolerant LTH and chilling-sensitive IR29) with contrasting responses to chilling stress were comparatively analyzed. Results revealed a differential constitutive gene expression prior to stress and distinct global transcription reprogramming between the two rice genotypes under time-series chilling stress and subsequent recovery conditions. A set of genes with higher basal expression were identified in chilling-tolerant LTH compared with chilling-sensitive IR29, indicating their possible role in intrinsic tolerance to chilling stress. Under chilling stress, the major effect on gene expression was up-regulation in the chilling- tolerant genotype and strong repression in chilling-sensitive genotype. Early responses to chilling stress in both genotypes featured commonly up-regulated genes related to transcription regulation and signal transduction, while functional categories for late phase chilling regulated genes were diverse with a wide range of functional adaptations to continuous stress. Following the cessation of chilling treatments, there was quick and efficient reversion of gene expression in the chilling-tolerant genotype, while the chilling-sensitive genotype displayed considerably slower recovering capacity at the transcriptional level. In addition, the detection of differentially-regulated TF genes and enriched *cis*-elements demonstrated that multiple regulatory pathways, including CBF and MYBS3 regulons, were involved in chilling stress tolerance. A number of the chilling-regulated genes identified in this study were co-localized onto previously fine-mapped cold-tolerance-related QTLs, providing candidates for gene cloning and elucidation of molecular mechanisms responsible for chilling tolerance in rice.

## Introduction

Chilling (0–15°C) stress is one of the major environmental factors limiting rice production in temperate and high altitude areas. During early growth stages, low temperature affects seed germination and seedling establishment. Later, during reproductive growth, chilling stress can inhibit proper microspore development, leading to a shortage of viable pollen during anthesis, and, eventually, to severe yield reductions [1], [2]. Consequently, improving chilling tolerance may lead to increased rice production.

Rice exhibits a high degree of genetic variability for chilling tolerance, with *indica* strains being more chilling sensitive than *japonica* rice, especially at the seedling stage [1], [3]. Genetic analysis indicates that chilling stress tolerance in rice is a quantitative trait, and a number of QTLs for chilling tolerance have been mapped to the rice genome [1], [4], [5], [6], [7], [8], indicating the possible involvement of a complex set of physiological and genetic mechanisms. Several major QTLs for chilling tolerance were recently fine-mapped to small chromosome regions using different mapping populations [9], [10], [11], [12], and one major QTL, *Ctb1*, from a cold-tolerant variety (Norin-PL8) has been cloned using a traditional map-based cloning strategy [13]. All of these results provide a foundation for further cloning of genes responsible for rice chilling tolerance.

Functional genomics analysis has revealed that complex regulatory networks are involved in chilling stress tolerance in plants. It is well known that the CBF/DREB-dependent response pathway plays an important role in low temperature tolerance during cold acclimation, and CBFs have been identified as the first wave of cold-induced genes [14]. Genome-wide gene profiling has determined that changes in gene expression occur in response to low temperature in a wide range of plant species. Using cDNA microarray analysis, a set of 121 genes were induced in a chilling-tolerant *japonica* rice by 24 h initial incubation at 10°C, and an “early response” regulatory network including ROS-bZIP1 was found to play a crucial role in short-term adaptive responses [15]. Further genome-wide gene profiling of rice in response to chilling stress revealed that several regulatory clusters, including bZIP factors acting on as1/ocs/TGA-like element-enriched clusters, ERF factors acting on GCC-box/JAre-like element-enriched clusters, and R2R3-MYB factors acting on MYB2-like element-enriched clusters, are involved in early chilling response, and oxidative signaling by H2O2 is at the center of the regulatory network [16].

A novel MYBS3-dependent pathway has recently been identified as essential for cold tolerance in rice. MYBS was found to repress the CBF–dependent cold signaling pathway. Molecular evidence indicates that CBF responds early, and MYBS late, to chilling stress, suggesting distinct pathways that function sequentially and complementarily to promote short- and long-term chilling stress adaptation in rice [17]. Additional but less-studied molecular pathways are also known to exist, and there are probably others that have not yet been uncovered.

Previous gene profiling experiments were performed using only a single genotype without comparing the transcriptomic differences between chilling-tolerant and chilling-sensitive varieties. Contrasting genotypes can serve as a powerful tool for understanding the physiological and molecular mechanisms of chilling tolerance in rice. In this study, parallel transcriptomic analysis in two rice genotypes with contrasting chilling-tolerant phenotypes was performed to identify and characterize novel genes involved in chilling stress tolerance in rice. The results of this study should contribute to our understanding of the evolution of environmental stress adaptation mechanisms and thereby assist efforts to improve rice tolerance using biotechnology and molecular breeding.

## Materials and Methods

### Plant Materials and Chilling Stress Treatment

Two rice cultivars that exhibit contrasting sensitivity to chilling were used in this study: the chilling-tolerant *japonica* genotype Li-Jiang-Xin-Tuan-Hei-Gu (LTH) and chilling-sensitive *indica* cultivar IR29. Mature non-dormant seeds were incubated at 30°C for 3 days prior to germination, and then sown in soil plates. LTH and IR29 were planted at the same spacing in the same plates; four plates each representing biological replicates were set up for both the chilling treatment and the control. Seedlings were allowed to grow to the S3 stage for 8 to 10 days at 29°C.

For the chilling stress treatments, S3-stage seedlings on soil plates were placed in a growth chamber (Beijing ZNYT, China) maintained at 4°C (±1°C) with a 12 h light/12 h dark photoperiod. This chilling stress treatment was suggested to be an effective method for evaluating chilling injury in cultivated rice at seedling stage [18], [19]. Control seedlings were grown under the same conditions except at 29°C. After 48 h of chilling treatment, seedlings on soil plates were moved to the control environment. Leaf samples from both chilled and control samples were collected with three biological replicates at 2, 8, 24, and 48 h time points during the course of the 48 h experiment, and then 24 h later, following recovery from chilling. All collected samples were snap-frozen in liquid nitrogen and stored at −70°C.

### Physiological Traits of the Two Genotypes under Chilling Stress

Membrane stability was measured using the procedure of reported before with minor modifications for rice leaf tissue [20]. Three replicates of 0.5 g fresh leaves were sampled from control and chilling-treated seedlings. After being cut into 1-cm pieces, the 0.5 g leaf samples were immersed in 20 mL distilled water in a test tube for 1 h with the help of a vacuum pump. After standing for 2 h at 25°C, water conductivity was measured. Leaf discs were then killed in the same solution by autoclaving, and total conductivity was measured at room temperature. Percent injury arising from each treatment was calculated from conductivity data using the equation: % injury = [(% L(t)−% L(c))/(100−% L(c))]×100), where % L (t) and % L(c) are percent conductivity for treated and control samples, respectively. Proline and MDA concentrations were measured according to the protocol of Shukla et al. [21]. Antioxidant enzyme activity, including SOD, POD, CAT, and GR, were determined following previously reported methods [22], and ascorbic acid and GSH concentration were determined as described in Qian et al. [23].

### Affymetrix GeneChip Hybridization and Data Analysis

Microarray hybridization was performed using an Affymetrix GeneChip Rice Genome Array (Affymetrix, Santa Clara, CA), which contain probes to query 51,279 transcripts from two rice cultivars, including 48,564 *japonica* and 1,260 *indica* transcripts. For microarray hybridization experiments, total RNA was extracted using TRIzol reagent and then purified and concentrated using an RNeasy MinElute Cleanup Kit (Qiagen). Preparation of cDNA and cRNA, array hybridization, and quality control checks were performed by Tianjin Biochip Corporation (Tianjin, China). Biotin-labeled cRNA was prepared using a GeneChip IVT Labeling Kit (Affymetrix, Santa Clara, USA); the cRNA fragments were then hybridized to the array for 16 h at 45°C using a GeneChip Hybridization Oven 640. After washing and staining with R-phycoerythrin streptavidin in a Genechip Fluidics Station 450, the arrays were scanned with a Genechip Scanner 3000 7G 4C. The scanned images were visually examined and then processed to generate raw data saved as CEL files using GCOS1.4 default settings. Array normalization was performed using dChip software. The original microarray data set has been deposited in NCBI’s Gene Expression Omnibus (GSE38023). Differentially expressed genes (DEGs) between the chilling stress sample and the control sample were identified by dChip software using a two-fold change threshold and in lower bound fold change method.

### Functional Classification and Prediction of Cis-acting Regulatory Elements

Functional enrichment/over-representation analysis was carried out using GOEAST (http://omicslab.genetics.ac.cn/GOEAST/index.php [24]) followed by manual adjustments. GO slim categories significantly overrepresented were calculated by a hypergeometric distribution with a cutoff level at 0.05. Using a Perl program and information downloaded from the PLACE *cis*-element database, *cis*-elements of genotype-specific response genes were identified from both strands of upstream 1-kb promoter sequences retrieved from rice genes. To determine over-representation of putative *cis*-regulatory elements between two groups of genes, *p*-values were calculated using a hypergeometric distribution, with *p*≤0.05 being used as the criterion for statistical significance for an identified *cis*-element.

### Quantitative Real-time PCR

Real-time PCR was performed using an ABI Prism 7900 Sequence Detection System (Applied Biosystems). Diluted cDNA was amplified using primers specific for the tissue-enriched genes and SYBR Green Master Mix (Applied Biosystems). Expression levels of tissue-enriched transcripts were normalized using an endogenous â-actin control. Each set of experiments was repeated three times, and the DDCT relative quantification method was used to evaluate quantitative variation. Primers used to amplify the selected genes are listed in Table S1.

## Results and Discussion

### Phenotypic and Physiological Performance of the Two Rice Genotypes Under Chilling Stress

**Figure 1:**
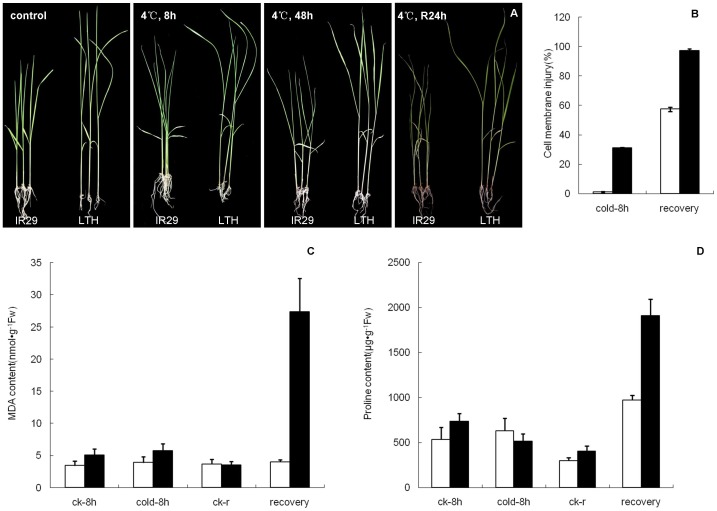
Phenotypes of two rice genotypes under chilling stress and subsequent recovery. (A) Comparison of seedlings of LTH and IR29 in control, treated at 4°C for 8 h, 48 h and recovery for 24 h after 48 h treatment. (B) Cell membrane injury of LTH and IR29 treated at 4°C for 8 h and recovery for 24 h after 48 h treatment. (C) and (D) MDA and proline content of LTH and IR29 treated at 4°C for 8 h and recovery for 24 h after 48 h treatment. LTH cultivar (white) and IR29 cultivar (black). Values are means of 3 replicates. Vertical bars indicate standard error.

Two rice genotypes with contrasting chilling tolerance were used in this study. Li-Jiang-Xin-Tuan-He-Gu (LTH) is widely identified as a chilling-tolerant (CT) *japonica* landrace variety [7], [25], [26], while IR29 is a chilling-sensitive *indica* cultivar [27]. To investigate physiological variation in chilling tolerance in the two genotypes, several indices of stress-induced effects were measured. LTH exhibited better chilling-stress tolerance and recovery ability than IR29, as observed by visual comparison of leaf rolling and wilting symptoms (Figure 1A). Compared with LTH, chilling-treated IR29 seedlings experienced more extensive cell membrane injury (relative electrolyte leakage) and exhibited higher MDA (malondialdehyde) concentration, especially after the 24 h recovery period following the 48 h chilling treatment (Figures 1B and 1C). There was no evident change of proline concentration detected in both genotypes under chilling stress compared with control, but significantly high levels of proline were detected in both cultivars after recovery (Figure 1D), indicating that proline may play an important role in the recovery process of rice plant under chilling stress.

Antioxidant enzyme activity and antioxidant concentration in seedlings subjected to chilling stress were also analyzed to assess the biochemical difference between the two genotypes. Slight changes in CAT, POD, and GR activity were observed after 8 h of chilling treatment in LTH compared with the control, while GR activity in IR29 increased nearly three-fold compared with the LTH cultivar. Both genotypes exhibited a remarkable increase in SOD activity after 8 h of chilling treatment compared with the control, with higher SOD activity in IR29 than LTH. Reduced glutathione (GSH) concentration after 8 h chilling stress and subsequent recovery was higher in IR29 than in LTH (Figure S1). These results indicate that certain antioxidants were involved in chilling stress response, which was in consistent with those reported previously [22]. Based on the different physiological traits measured in this study and previously reported evidences, LTH and IR29 clearly differ in their response to chilling stress.

### Genome-wide Gene Expression of Two Contrasting Rice Genotypes Under Chilling Stress Using an Affymetrix Rice Genome Array

To investigate chilling-induced alterations in gene expression of the two genotypes in this study, an Affymetrix rice genome array was used. Differentially expressed genes (DEGs) under chilling stress and subsequent recovery conditions were identified using the combined criteria of two-fold or more change and a significant *t*-test (*p*<0.05) based on three biological replicates. We found 8,484 genes differentially regulated in either of genotypes under stress and recovery compared with the control. Among them, 7,158 DEGs were detected during at least one chilling-stress time point, and 3,230 DEGs were identified under recovery condition.

**Figure 2:**
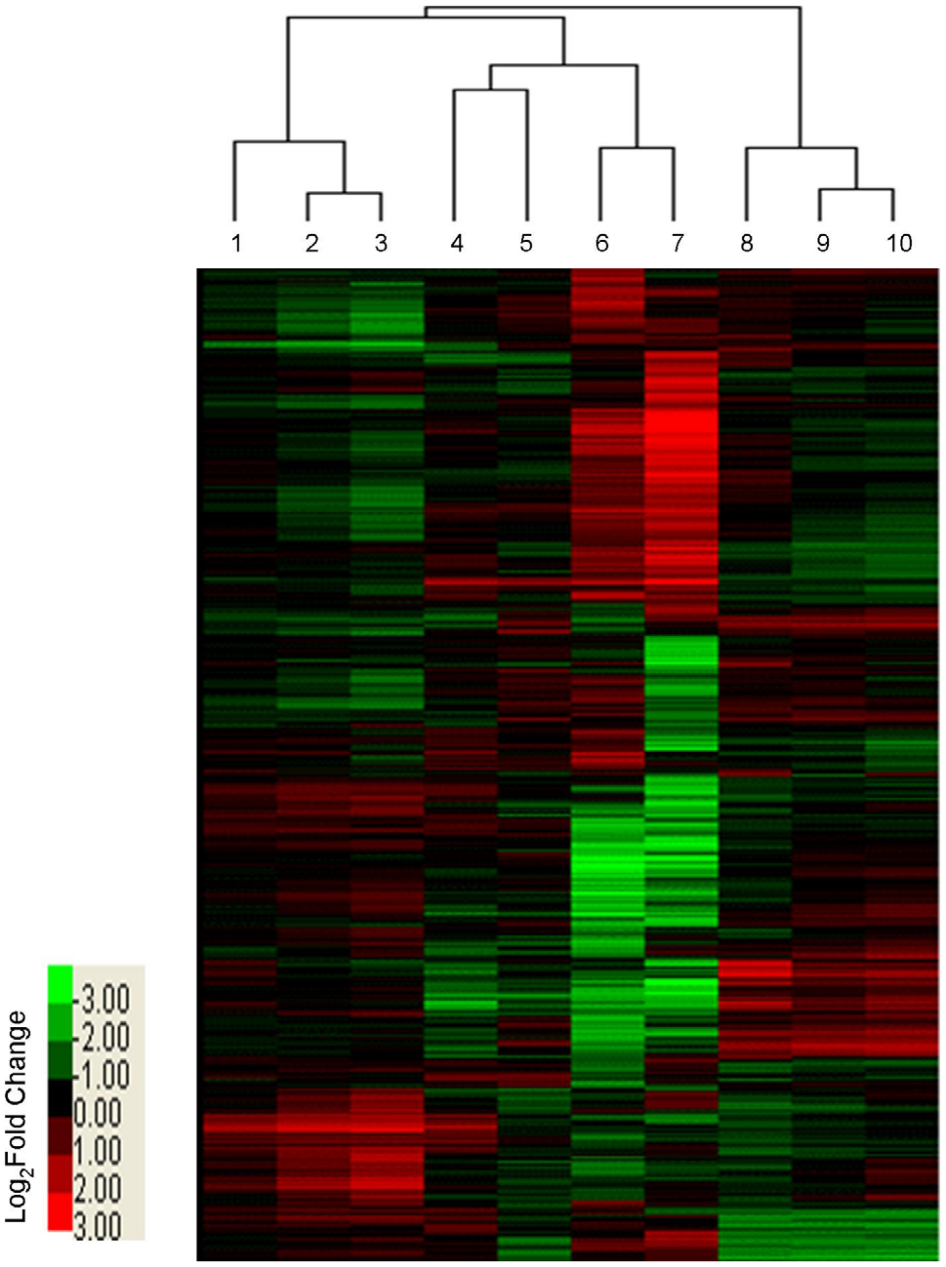
Hierarchical cluster analysis of all DEGs in LTH and IR29. These include 8484 DEGs at 2, 8, 24, and 48 h time points during chilling-stress treatments and subsequent 24 h recovery. The median ratio (stressed/control) was log (base 2)-transformed and subjected to linkage hierarchical clustering. 1, 2, 3 indicate LTH at 8, 24, and 48 h chilling-stress time points, respectively; 4 and 5 indicate LTH and IR29 at 2 h chilling stress, respectively; 6 and 7 indicate LTH and IR29 during 24 h recovery, respectively; 8, 9, and 10 indicate IR29 at 8, 24, and 48 h chilling-stress time points, respectively.

To assess similarities and differences between the low-temperature transcriptomes of LTH and IR29, cluster analysis of all 8,484 DEGs was performed using MeV (http://www.tm4.org/mev/). As shown in Figure 2, the 2 h chilling-stressed samples of both genotypes clustered together, but at all later chilling and recovery time points, LTH and IR29 formed separate clusters. In addition, the recovery samples of these two genotypes were grouped into a single cluster. These results clearly indicate that the transcriptomic responsiveness to chilling stress in LTH and IR29 can be divided into two phases: an early response phase (2 h chilling stress) and late response phase (8, 24, and 48 h chilling stress), consistent with previous reports on chilling stress gene profiling [15], [16].

The chilling-induced DEG profiles detected using the microarray were confirmed for 24 selected genes using quantitative RT-PCR analysis (Figure S2). In total, 240 comparisons were made, as the expression of each gene was monitored at five separate chilling treatment time points compared with the control in both LTH and IR29 (24*5*2). Gene expression profiles identified by the microarray experiments exhibited a high degree of similarity (*r* = 0.892) to those obtained from the quantitative RT-PCR analyses, thus confirming the reliability and robustness of the microarray data.

### Intrinsic Transcriptome Differences of LTH and IR29 Prior to Chilling Stress

Phenotypic differences resulting from gene expression variation have been observed in different ecotypes of *Arabidopsis thaliana* [28]. To explore the intrinsic difference of gene expression to chilling stress, gene expression levels in LTH and IR29 under control conditions were analyzed; 286 and 280 genes were up- and down-regulated, respectively, in LTH compared with IR29 under normal growth conditions. Previous studies have suggested that the highly constitutive gene expression prior to abiotic stress treatment might represent a constitutive tolerance in tolerant genotypes [29], [30], [31], [32]. As shown in Table S2, the genes with higher basal expression in LTH were functionally enriched in metabolism, stress response, signal transduction, transcription regulation, and redox regulation based on GO analysis. Based on their expression under chilling stress, these genes can be classified into several groups as follows.

The largest group comprises genes unresponsive to chilling stress in either genotype. This includes 154 genes that were heavily represented in LTH, such as those encoding glutathione S-transferase, oxidoreductase and thioredoxin, which promote chilling tolerance by maintaining cell redox homeostasis [33], [34], [35], [36]. A few stress-responsive genes encoding calcium homeostasis regulator CHoR1, death-associated protein kinase 1, four LRR-containing proteins, and four F-box domain-containing proteins also belong to this group. Even though not all of the genes in this group are involved in chilling-stress response, the higher basal expression levels of those genes that are related to stress-responsive and redox regulation may be responsible for the intrinsic tolerance to chilling stress found in LTH.

The second group consists of 27 genes induced in LTH or IR29 during at least one chilling-stress time point. Among these, there were six genes encoding transcription factors, two genes encoding short-chain dehydrogenase/reductase (SDR) family proteins, and three stress-responsive genes encoding senescence-associated protein DIN1, hairpin-induced protein 1, and polygalacturonase inhibitor 1, indicating their positive role in chilling-stress responsiveness.

Among the genes that were enriched in LTH compared with IR29 under control conditions, there were 77 genes found to be repressed exclusively in LTH during at least one time point in the chilling stress treatment. Excluding 43 genes of unknown function, they were functionally prevalent in redox regulation, metabolism, and stress response and transport, including genes encoding several peroxidases, oxidoreductases, wall-associated kinase 3, and receptor-like kinase ARK1AS. These genes might have a role in the negative regulation of chilling stress tolerance or LTH-specific responsiveness to chilling stress.

A large number of genes involved in transcription regulation, transport, and metabolism were found to be repressed in LTH compared with IR29 under control conditions; these genes included those encoding two heat shock proteins (HSP), one heat shock transcription factor (HSF), a photosystem II D2 protein, and a set of protein kinase domain-containing proteins (Table S3). HSP and HSF play a central role in heat shock response in plants and also in other organisms [30], [37], but the role of these heat-shock-related proteins in chilling stress responsiveness of plants still needs to be analyzed.

### Genotypic Differences in Transcriptomic Response to Chilling Stress

**Figure 3:**
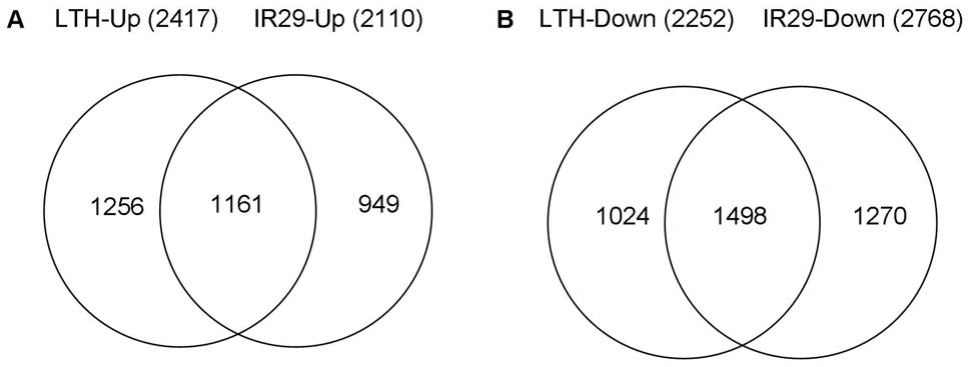
Venn diagram of up- and down-regulated genes in LTH and IR29 under chilling stress conditions. (A) Up-regulated genes; (B) down-regulated genes.

Among the 7158 DEGs found in both genotypes under chilling stress, there were 4939 and 4878 genes showing differential expression in LTH and IR29, respectively. Of the up-regulated genes, 1256 were exclusive to LTH, 949 were found only in IR29, and 1161 were identified in both genotypes (Figure 3A). Of the down-regulated genes, 1024 and 1270 were identified as LTH- and IR29-specific, respectively, and 1498 were repressed in both genotypes (Figure 3B).

A comparison of the kinetics of gene expression pattern changes in LTH and IR29 revealed that chilling stress induced a continuous increase in DEGs; the number of up-regulated genes clearly exceeded that of down-regulated genes in LTH at each chilling treatment time point, whilst chilling stress caused more genes to be repressed in IR29 over the course of the stress treatment (Table S4). These results may reflect the distinct nature of chilling stress responsiveness in tolerant and sensitive genotypes. As we did based using the previously-mentioned cluster analysis results, we classified genes with expression level changes at 2 h as early response (ER) genes, and grouped those with altered expression at 8, 24, and 48 h as late response (LR) genes.

### Early Response Genes of LTH and IR29 Under Chilling Stress

As shown in Table S4, there were 2044 LTH and 2080 IR29 DEGs detected in the ER phase. In LTH at 2 h of chilling treatment, 1166 genes were up-regulated and 878 genes were down-regulated, while 923 induced and 1157 repressed genes were found in IR29. In the up-regulated gene set, there were 554, 568, and 330 induced genes identified to be common-, LTH-specific, and IR29-specific, respectively, after 2 h chilling stress (Tables S5, S6, and S7).

**Figure 4:**
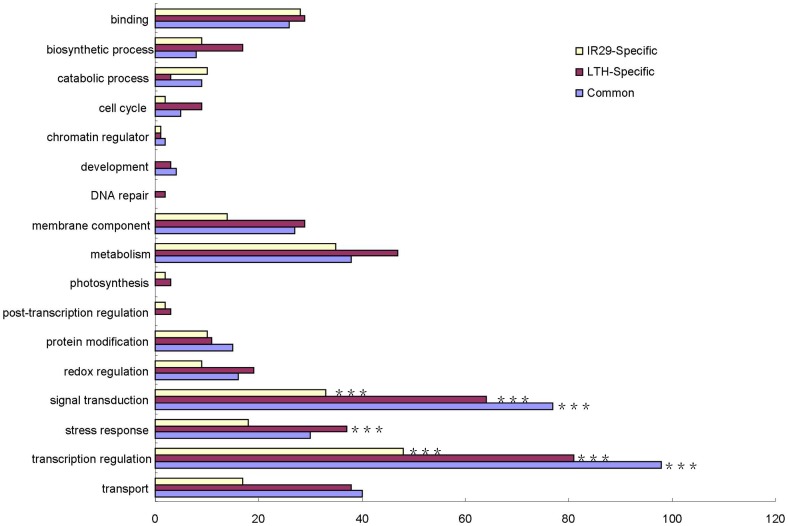
GO slims of functional categorization of genes at the 2 h chilling-stress time point. The genes found to be commonly- or genotype-specifically-induced in LTH and IR29. Genes with unknown function are not included. Bars show numbers of common (blue), LTH-specific (purple) and IR29-specific (yellow) induced genes. GO slim categories significantly overrepresented are calculated by a hypergeometric distribution and indicated by ***for P≤0.05.

Broad biological functions of the 2 h chilling-induced genes in both genotypes were comparatively analyzed in terms of GO enrichment. A large proportion (17.7% common, 14.3% LTH-specific, and 14.5% IR29-specific) of genes induced after 2 h chilling stress were involved in transcriptional regulation (Figure 4). Most of these genes encode different transcription factors, including AP2/EREBP, MYB, HSF, and NAC proteins. In addition, a number of genes (77 common, 64 LTH-specific, and 33 IR29-Specific) related to signal transduction were identified at the ER phase of chilling stress (Figure 4). These include genes encoding calcium-dependent protein kinases, calcium-transporting ATPases, calmodulin, mitogen-activated protein kinase 1, protein phosphatase 2C family proteins, and serine/threonine-protein kinases. All these genes have previously been determined to be functionally involved in signal transduction pathways triggered in plants by abiotic stresses [38], [39], [40], [41], [42]. These results are consistent with a recent report revealing that the common early phase of transcriptomic responses to different abiotic stresses is characterized by alterations of genes related to signaling cascades such as receptor kinases, transcription factors, and components of calcium signaling [43].

LTH-specific induced transcripts are of particular interest as their functional annotation may provide insights into possible molecular mechanisms of chilling tolerance in rice. A number of genes with a wide range of functions were identified as LTH-specifically induced by early chilling stress. Most of these genes encode proteins involved in stress response and signaling transduction, such as those encoding acyl-coenzyme A oxidase 4 (*OsACX4*), S-adenosyl-L-methionine decarboxylases, and those homologs of the barley mildew resistance locus O (MLO) proteins (LOC_Os02g35490, MLO-like protein 1; LOC_Os10g39520, MLO-like protein 10). Among these, acyl-CoA oxidases catalyse the first step in fatty acid beta-oxidation, the pathway responsible for lipid catabolism and plant hormone biosynthesis [44]. In our study, *OsACX4* was only up regulated at 2 h of chilling treatment in LTH, implying a role in the early signaling pathway of chilling stress. MLO-like proteins are involved in modulation of pathogen defense and the cell cycle, and their activity seems to be regulated by Ca^2+^-dependent calmodulin binding [45]. The gene *OsSAMDC*, which encodes an S-adenosyl-L-methionine decarboxylase (SAMDC) involved in polyamine biosynthesis, has been suggested for use as a molecular marker in the identification of rice tolerance to low temperature [46]. It has also been reported that *SAMDC* is specifically induced in cold-stressed potato [47], and *SAMDC* over-expression enhanced cold tolerance in transgenic tobacco plants [48]. Two genes encoding SAMDC were highly induced exclusively in LTH under chilling stress, confirming that *SAMDC* could be used as an induced expression marker for chilling tolerance.

Histones are basic proteins packaging DNA into nucleosomes, and histone gene expression is tightly correlated with the cell cycle and cell proliferation [49]. It has been reported that histone genes are repressed by abiotic stresses such as cold [50], drought, and salinity [51], [52]. Strikingly, a set of nucleosome core histone (H2A, H2B, H3, H4) genes were found to be down-regulated exclusively in LTH under chilling stress in the present study (Table S6), that implying these core histone genes are involved in chilling tolerance. Among these histone genes, H2A.Z, encoding a particular histone variant, has been found to play a crucial role in temperature perception through DNA-nucleosome fluctuations [53]. Specific repression of H2A.Z in LTH under chilling stress demonstrates its temperature-dependent regulation.

Interestingly, we detected a number of genes functionally involved in carbohydrate metabolism down-regulated by ER-chilling specifically in IR29 (Table S7). These included a set of eight genes encoding UDP-glucoronosyl and UDP-glucosyl transferase (UGT) family proteins, three soluble starch synthase genes, and four sugar transporter genes. UGTs are involved in sugar metabolism and transport [54] and in detoxification [55], and starch synthase genes are involved in starch synthesis metabolism [56]. Repressions of these genes indicate carbohydrate metabolism and transport was specifically reduced in IR29 at low temperature.

Previous studies have revealed that the general dynamics of plant stress response can be classified into several phases, such as an early phase (early alarm phase), a late phase including an acclimation phase (middle phase), and a resistance phase [43], [57], [58]. In the present study, we determined that a large proportion of DEGs common to both genotypes were induced during early phases of chilling stress response, which suggests that they are general and crucial components involved in chilling stress signaling. In addition, a number of DEGs altered during the ER phase were characterized by genotype-dependent expression patterns, revealing intrinsic transcriptomic responses to early chilling stress.

### Differential Molecular Responses Under Late Phases of Chilling Stress in the Two Rice Genotypes

**Figure 5:**
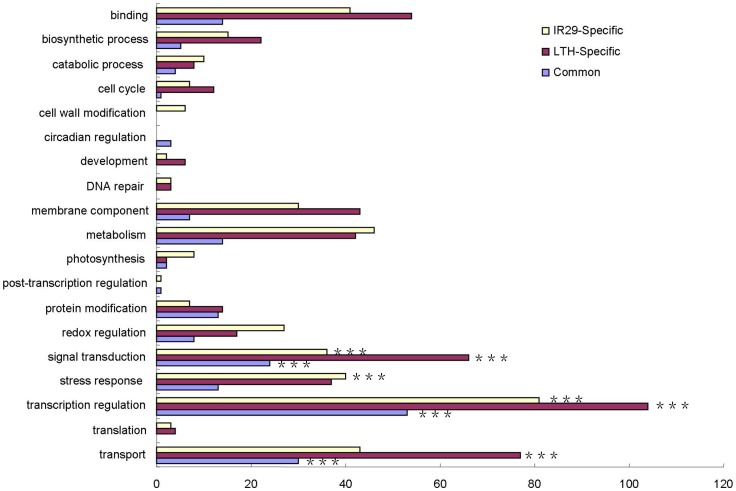
GO slims of functional categorization of the genes during the late response phase. The genes found to be commonly- or genotype-specifically-induced in LTH and IR29 during chilling stress of 8, 24, and 48 h. Bars show numbers of common (blue), LTH-specific (purple) and IR29-specific (yellow) induced genes. GO slim categories significantly overrepresented are calculated by a hypergeometric distribution and indicated by ***for P≤0.05.

Based on the results of cluster analysis, we classified as late response genes only those differentially regulated in both genotypes after 8 h of chilling stress. In total, there were 1082 and 967 genes detected as LR up-regulated genes in LTH and IR29, respectively. Among these, 299 genes were commonly induced in both genotypes, 783 were induced only in LTH, and 668 were up-regulated specifically in IR29. There were 549, 731, and 790 genes identified as common, LTH-specific, and IR29-specifically repressed during LR-chilling stress, respectively (Tables S8, S9, and S10). The most prevalent functional categories for the LR-induced genes in both genotypes were transcription regulation, transport, signal transduction, binding function, and membrane component (Figure 5). These results show that most of the LR genes were genotype-specific, implying that chilling stress responsiveness during the LR phase is clearly diversified at the transcriptome level in different genotypes.

With respect to the commonly-regulated genes, a set of nine genes encoding heat shock-related proteins (HSPs), including two heat shock cognate 70 kDa proteins and two heat shock factors (HSFs), were repressed in both genotypes (Table S8). Extensive evidence indicates that heat shock-related genes in plants are involved in abiotic stress response, especially to extreme environmental temperature [59]. Down-regulation of these HSPs and HSFs suggests they might play a different role in rice plants at low temperature compared with their positive regulation in response to heat.

Auxin plays an important role in plant response to cold stress [60]. A number of auxin-related genes encoding auxin/IAA family proteins, auxin-induced proteins, auxin response factors, and auxin-associated proteins were regulated by late phase chilling stress in both genotypes (7 common, 8 LTH-specific, and 12 IR29-specifically regulated). This observation indicates an interaction between auxin signaling and chilling stress response involving both common and divergent mechanisms.

Leucine-rich repeat proteins constitute a large gene family and play important and functionally diverse roles in plant growth and development [61]. Ten and eight LRR genes were significantly up-regulated by LR-chilling stress in LTH and IR29, respectively. These results imply that LRR proteins could have an important role in chilling stress response, probably by maintaining membrane stability.

Aquaporin is a membrane protein that facilitates water transport across biological membranes. Aquaporin genes have been found to be involved in molecular responses to abiotic stresses [62]. Three genes (Os07g0448400, Os02g0666200, and Os01g0975900) encoding aquaporin were down-regulated in LTH during LR chilling stress, implying that chilling-induced repression of these aquaporin gene expressions could result in reduced membrane water permeability and thus maintain water homeostasis in rice plants responding to chilling stress.

Many physiological studies have documented that photosynthesis in various crop plants is greatly inhibited by low temperature [63], [64], but we detected seven photosynthesis-related genes up-regulated exclusively in IR29 during LR chilling stress (Table S10). Up-regulation of these genes in the chilling-sensitive genotype might be due to chloroplasts adapting to the changed conditions, or simply be a genotype-specific response to long-duration chilling stress.

Strikingly, five genes corresponding to 5′-adenylylsulfate reductase 2, NADPH: quinone oxidoreductase, NmrA-like family protein, oxidoreductase, and thioredoxin family proteins were highly induced in LTH (Table S9), but evidently repressed in IR29 under LR-chilling stress (Table S10). These genes are functionally involved in cell redox homeostasis according to GO analysis. There is extensive evidence that different antioxidant compounds, including oxidoreductase and thioredoxin, contribute to general redox homeostasis in plant cells undergoing developmental or environmental stress [55], [65]. Differences in expression or activity levels of antioxidant enzymes under stress have been found to be genotype-dependent; these differences were specific to either tolerant or sensitive genotypes [22], [66], suggesting that these genes with differential regulation in LTH and IR29 under chilling were involved in stress tolerance, or, that there may simply be genetic differences in ROS regulatory pathways.

Plants respond to stress by progressively adjusting their transcriptome with sustained, transient, early- and late-responsive gene expression alterations [43]. In contrast to the ER phase, most DEGs were found to be genotype-specifically regulated in the late phase of chilling stress treatment, demonstrating that the differential transcriptome response to LR-chilling stress between LTH and IR29 was based on genetic differences.

### Differential Transcriptomic Responses during Recovery in the Two Genotypes

A total of 3230 genes were found to be differentially-regulated in LTH and IR29 during 24 h recovery conditions. In LTH, there were 219 and 226 genes up- and down-regulated, respectively, of which 224 genes were exclusively regulated during the recovery process. A large number of differentially-regulated genes were observed in IR29; we identified 1173 and 1834 genes up- and down-regulated, respectively, during recovery. Among these genes, 1685 were only induced or repressed under recovery conditions (Table S11).

A total of 232 genes were co-regulated in both genotypes under recovery conditions compared with those under control conditions. GO categorization showed that commonly up-regulated genes during recovery were enriched in metabolic, oxidation-reduction, and stress-response processes, indicating their general role in recovery mechanisms of rice plants after chilling stress.

The observation that the greatest number of DEGs was detected exclusively in IR29 at recovery indicates a slow recovery process of global gene expression alterations caused by chilling stress. As expected, many of the DEGs in IR29 were GO-prevalent in cellular (293 genes up-regulated, 424 down-regulated), metabolic (352 up-regulated, 503 down-regulated), biosynthetic process (103 up-regulated, 142 down-regulated), and oxidation-reduction (85 up-regulated, 122 down-regulated) processes. In contrast, LTH-specific DEGs during recovery were only enriched in stress-response (7 genes up-regulated) and metabolic (36 genes down-regulated) processes; these genes include those encoding a 17.4 kDa class I heat shock protein, a dehydrin, and two late embryogenesis-abundant proteins. The most dramatic gene expression differences between chilling-tolerant and chilling-sensitive genotypes were detected during recovery conditions, suggesting that tolerant genotypes activate mechanisms that allow quicker and more efficient recovery in gene expression after chilling stress.

### Genotype-dependent Chilling-stress-responsive Transcription Factors

We detected 542 transcription factor (TF) genes differentially regulated in LTH and IR29 under chilling stress and recovery (Table S12). There were 267 up-regulated and 72 down-regulated TF genes in LTH identified during at least one chilling stress or recovery time point compared with control conditions; in IR29, 277 and 163 TF genes were up- and down-regulated, respectively, compared with those under control conditions. Although most TF genes were commonly regulated under chilling stress, we detected a subset of TF genes uniquely induced or repressed in either LTH or IR29 by low temperature and subsequent recovery.

The differentially-expressed TF genes categorized by family were summarized in Table S13. The vast majority were up-regulated. Most were differentially expressed in the two genotypes at 2 h in the chilling treatment, demonstrating that many TF genes were involved in early response by rice plants to chilling stress. In addition, 27 WRKY, 7 Tify, and 13 GRAS TF genes were induced in both genotypes, showing their positive regulatory roles in rice plant response to low temperature. Under recovery conditions, 15 and 9 TF genes were up- and down-regulated, respectively, in LTH, including 6 NAC, 7 MYB, and 3 Tify genes. In IR29, 69 induced and 108 repressed TF genes were identified during the recovery period. Of these, 114 genes (35 induced and 79 repressed) were regulated exclusively in IR29 during recovery, suggesting that many additional regulatory genes were involved in the recovery process in the chilling-sensitive cultivar IR29.

The AP2/EREBP family comprises a large group of plant-specific transcription factors and is involved in abiotic stress response [67]. In this study, we found 43 AP2/EREBP genes differentially-regulated at low temperature, of which 27 were commonly regulated in both genotypes. There were 6 and 11 AP2/EREBP genes exclusively induced in LTH and IR29, respectively, by chilling stress (Table S12). *OsCBF1* (Os09g0522000), *OsCBF2* (Os06g0127100), and *OsCBF3* (Os02g0677300*/*Os09g0522200) were highly up-regulated in both genotypes, which is consistent with previous reports that these DREBs/CBFs contribute to low temperature stress response by regulating many transcriptomic and metabolic changes [16], [68], [69]. Interestingly, two additional copies (Os08g0408500 and Os04g0572400) of *CBF1* were uniquely induced during the LR phase of chilling stress in LTH and IR29, respectively, suggesting these duplicated *CBF1* genes are associated with genotype-specific responses to chilling stress. Among the differentially-regulated AP2/EREBP genes, two *RAV1* genes were specifically induced in LTH and IR29 (LOC_Os01g04750 in LTH at the ER phase and LOC_Os01g49830 in IR29 at the LR phase). RAV1 is thought to play an important role in the cold stress response pathway, most likely as a component of the CBF regulon [70], and this gene is also closely associated with leaf maturation and senescence [71]. Unique expression patterns of different *OsRAV1* gene copies in specific genotypes under chilling stress might indicate a distinct role in genotype-dependent responsiveness to chilling stress.

Rice has at least four DREB2 homologs, among which *OsDREB2B* was found in one study to be highly induced after 24 h chilling stress, and its expression was regulated by alternative splicing that generates both functional and nonfunctional transcripts. Transgenic *Arabidopsis* plants over-expressing the functional form of *OsDREB2B* displayed improved tolerance to abiotic stresses [72]. In the present study, *OsDREB2B* (Os05g0346200) was up-regulated in LTH at 8 h and IR29 at 24 h after onset of chilling stress, indicating that *OsDREB2B* functions as a key gene in late response to chilling stress in both genotypes, but with different initiation times.

More than half of the 103 WRKY TF genes that have been identified in the rice genome have been shown to be transcriptionally regulated under conditions of biotic and/or abiotic stress [73], [74]. In this study, a set of WRKY genes were found to be chilling-regulated in both genotypes, of which 20 were commonly induced, 2 were up-regulated exclusively in LTH, and 8 were up-regulated exclusively in IR29. Three chilling-induced WRKY genes (Os05g0343400, Os01g0246700, and Os01g0826400) in both genotypes were also observed to be induced by chilling stress in a *japonica* rice variety [16]. The remaining WRKY TF genes are reported here for the first time to be induced by chilling stress; further analysis for functional identification of these WRKY genes is needed.

We detected 41 MYB and MYB-related TF genes differentially expressed by low temperature in both genotypes, 23 of which were commonly regulated, and 13 and 8 of which were regulated exclusively in LTH and IR29, respectively. Thirteen of these MYB genes (Os05g0449900, Os11g0684000, Os02g0624300, Os01g0841500, Os09g0538400, Os05g0140100, Os10g0478300, Os04g0517100, Os01g0298400, Os05g0195700, Os02g0641300, Os08g0144000, and Os01g0187900) have been found to be regulated by chilling or H_2_O_2_ stress in *japonica* rice [16], suggesting these MYB TF genes are functionally involved in rice chilling stress response. *OsMYB15* (Os02g0624300) was chilling-induced in both genotypes; its homologous gene in *Arabidopsis* (*MYB15*, At3g23250) is involved in negative control of the cold tolerance pathway [75], revealing their diverse functional regulatory roles in rice and *Arabidopsis*. We detected two MYB genes, *OsMYBS3* (Os10g0561400) and *OsMYB3R2* (Os01g0841500), uniquely induced in the late phase of chilling stress in LTH. *OsMYBS3* has been found to repress the well-known DREB1/CBF-dependent cold signaling pathway in rice, and it responds more slowly to cold stress than *DREB1*, implying an additional novel pathway that controls cold adaptation in rice [8]. Over-expression of *OsMYB3R2* may significantly improve cold tolerance by mediating the alteration in cell cycle and ectopic expression of stress genes in rice [76]. All of these results suggest that *OsMYBS3* and *OsMYB3R2* play key roles in the regulatory pathway of chilling stress tolerance in the chilling-tolerant rice LTH.

### Detection of Highly-enriched Cis-elements in ER- and LR- Chilling-induced Genes

*Cis*-acting elements/motifs act as activators or repressors in gene transcription by allowing TF recognition and binding. We identified over-represented motifs in the promoter regions of the ER- and LR-induced genes in both genotypes. As shown in Table S14, the top three *cis*-elements for ER-induced genes were CANNTG, CACT, and AAAG, which function as unique binding motifs for CBF genes [77], mesophyll-specific expressed genes [78], and Dof genes [79], respectively. In comparison, the top three motifs detected among LR-induced genes were CACT, GATA, and AAAG (Table S15). The GATA core sequence is involved in light-regulated expression of nuclear genes [80]. The highly-enriched CBF binding motifs in ER chilling-induced genes demonstrate that CBF gene regulation is initiated during the early phase of chilling stress and plays a crucial role in chilling stress response in rice plants. The remaining motifs mentioned above are functionally related to photosynthesis and light regulation, implying that the chilling-stress responsiveness of plants is strongly associated with the presence of light and photosynthetic activity.

### DEGs Mapped to the Previously Identified Chilling-related QTL Intervals

Based on the Gramene QTL database (www.gramene.org/db/), a total of 37 QTLs related to chilling tolerance in rice have been identified. We located 445 genes differentially-regulated by chilling stress on 21 of these identified QTL intervals. Among them, the QTLs *qCTB1*, *qCTS1*, *qCTS4-3*, *qCTS6-2*, *qCTS8-1*, and *qPSST-9* had the greatest number of co-localized DEGs with 36, 28, 37, 80, 65, and 20 genes, respectively (Table S16).

The QTL *qCTS6-2* is the most important QTL related to chilling-induced wilting tolerance in rice at seedling stage [4]. Eight DEGs were co-localized on the *qCTS6-2* 0.9 cM interval; among them, a cluster of genes encoding one AP2 TF, two phospholipases D, one LRR protein, and a CBL-interacting serine/threonine-protein kinase were evidently induced by LR-chilling stress in either LTH or IR29. A major QTL associated with cold-induced necrosis and wilting tolerance, designated as *qCTS12a* [81], was co-localized with four DEGs, including two isoflavone reductases, one 3-hydroxyisobutyryl-coenzyme A hydrolase, and one non-protein coding transcript, which were highly repressed during LR-chilling stress.

The major QTL for chilling tolerance at the booting stage, *qCTB7*, was fine-mapped by Zhou et al. [11]. Four genes on the *qCTB7* interval were found to be differentially regulated by chilling stress in this study; a gene encoding pre-mRNA processing protein PRP39 was highly induced only in LTH, while two genes encoding hydrolysis and chlorophyll a–b binding protein 7 were strongly repressed exclusively in IR29. These DEGs can serve as functional candidate genes for the identification of a QTL for chilling tolerance. By combining further functional identification and QTL fine mapping, the co-localized DEGs detected in this microarray analysis may provide the basis for gene cloning and elucidation of the molecular mechanisms responsible for chilling tolerance in rice.

### Conclusions

In the present study, comprehensive gene expression using an Affymetrix rice genome array revealed a genetic difference in adaptation to low temperature and a diverse global transcription reprogramming between two rice genotypes under chilling stress and subsequent recovery conditions. Firstly, the chilling tolerant LTH showed a different constitutive gene expression profile compared to the chilling-sensitive IR29. Second, the dominant change in gene expression at low temperature was up-regulation in the chilling-tolerant genotype and down-regulation in the chilling-sensitive genotype. Early responses to chilling stress common to both genotypes featured up-regulated genes related to transcription regulation and signal transduction, while functional categories of LR-chilling regulated genes were clearly diverse with a wide range of functional adaptations in two genotypes. Thirdly, at the end of the chilling treatments, there was quick and efficient reversion of gene expression in the chilling-tolerant LTH, while the chilling sensitive IR29 displayed considerably slower recovery capacity at the transcriptional level. Finally, analysis of differentially-regulated TF genes and enriched *cis*-elements demonstrated that multiple regulatory pathways, including CBF and MYBS3 regulons, are involved in chilling stress tolerance.

## Supporting Information

Figure S1 **Activity of ROS-scavenging enzymes and antioxidants concentration in seedlings of the two rice genotypes subjected to cold stress.**
(PPT)

Figure S2 **Comparison of microarray and quantitative RT-PCR assay data, based on the ratio between sample and control (S/C) in LTH and IR29.**
(PPT)

Table S1 **List of gene primers used for quantitative RT-PCR.**
(DOC)

Table S2 **LTH-enriched genes compared with IR29 under control conditions.**
(XLS)

Table S3 **LTH-repressed genes compared with IR29 under control conditions.**
(XLS)

Table S4 **Summary of differentially expressed genes under chilling stress and recovery conditions.**
(DOC)

Table S5 **Common DEGs in LTH and IR29 after 2 h chilling stress.**
(XLS)

Table S6 **LTH-specific DEGs during ER chilling stress.**
(XLS)

Table S7 **IR29-specific DEGs during ER chilling stress.**
(XLS)

Table S8 **Common DEGs in LTH and IR29 during LR chilling stress.**
(XLS)

Table S9 **LTH-specific DEGs during LR chilling stress.**
(XLS)

Table S10 **IR29-specific DEGs during LR chilling stress.**
(XLS)

Table S11 **DEGs in LTH and IR29 on recovery.**
(XLS)

Table S12 **Differentially-regulated TF genes in LTH and IR29 under chilling stress.**
(XLS)

Table S13 **Transcription factor genes differentially regulated in LTH and IR29 during chilling stress treatments.**
(DOC)

Table S14 ***Cis*** **-element analysis for the induced genes during ER chilling stress.**
(DOC)

Table S15 ***Cis*** **-element analysis for the induced genes during LR chilling stress.**
(DOC)

Table S16 **List of differentially-regulated genes mapped to previously detected QTL intervals.**
(XLS)
