# Supplementary material for: Comparative Transcriptome Profiling of Chilling Stress Responsiveness in Two Contrasting Rice Genotypes
Source: PLoS One. 2012 Aug 17;7(8):e43274. doi: 10.1371/journal.pone.0043274 (PMC3422246; doi:10.1371/journal.pone.0043274)
Supplement: Figure S1 — Activity of ROS-scavenging enzymes and antioxidants concentration in seedlings of the two rice genotypes subjected to cold stress. (PPT) [file pone.0043274.s001.ppt]

## Slide 1
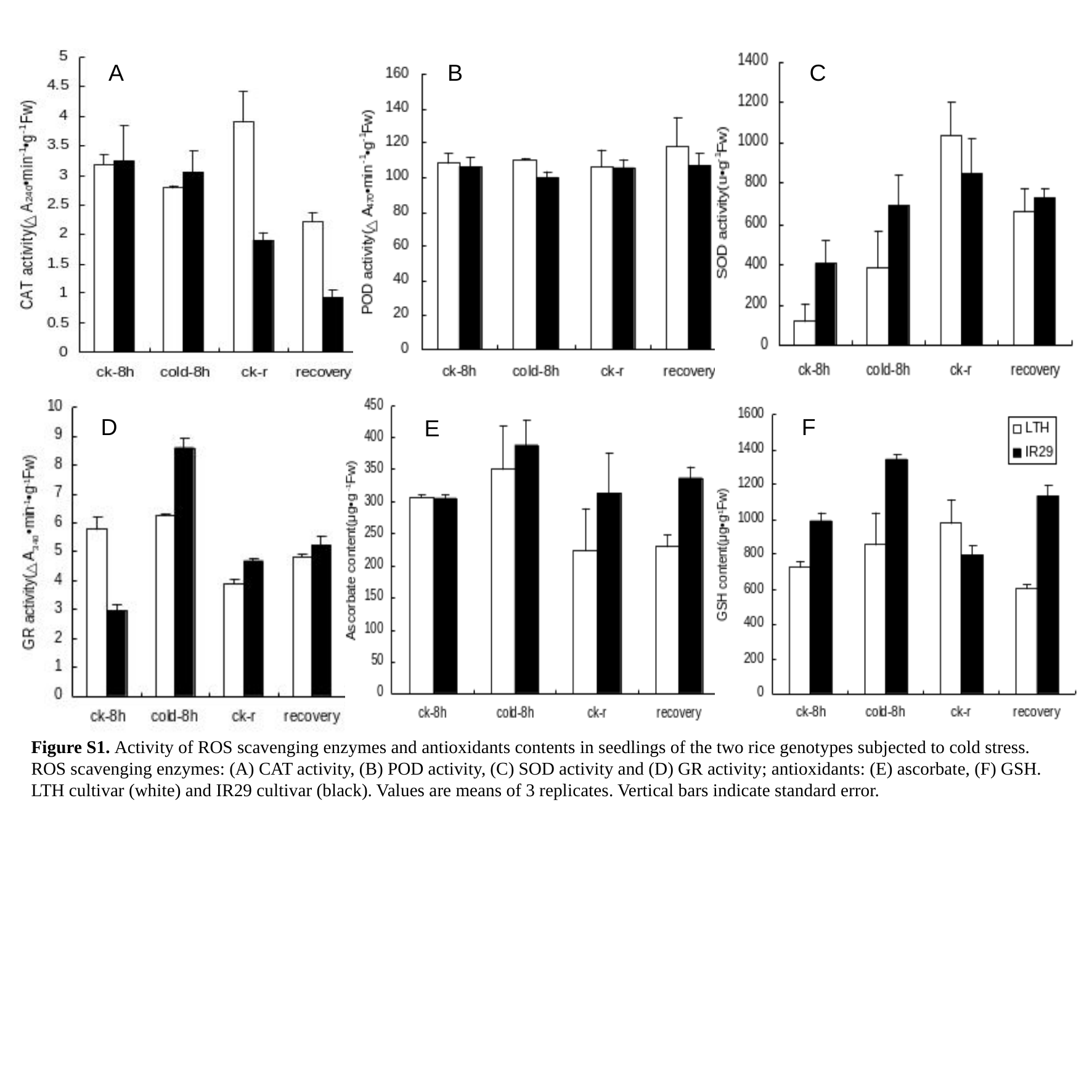

A
B
C
D
F
E
Figure S1. Activity of ROS scavenging enzymes and antioxidants contents in seedlings of the two rice genotypes subjected to cold stress. ROS scavenging enzymes: (A) CAT activity, (B) POD activity, (C) SOD activity and (D) GR activity; antioxidants: (E) ascorbate, (F) GSH. LTH cultivar (white) and IR29 cultivar (black). Values are means of 3 replicates. Vertical bars indicate standard error.
