# Supplementary material for: Comparative Transcriptome Profiling of Chilling Stress Responsiveness in Two Contrasting Rice Genotypes
Source: PLoS One. 2012 Aug 17;7(8):e43274. doi: 10.1371/journal.pone.0043274 (PMC3422246; doi:10.1371/journal.pone.0043274)
Supplement: Figure S2 — Comparison of microarray and quantitative RT-PCR assay data, based on the ratio between sample and control (S/C) in LTH and IR29. (PPT) [file pone.0043274.s002.ppt]

## Slide 1
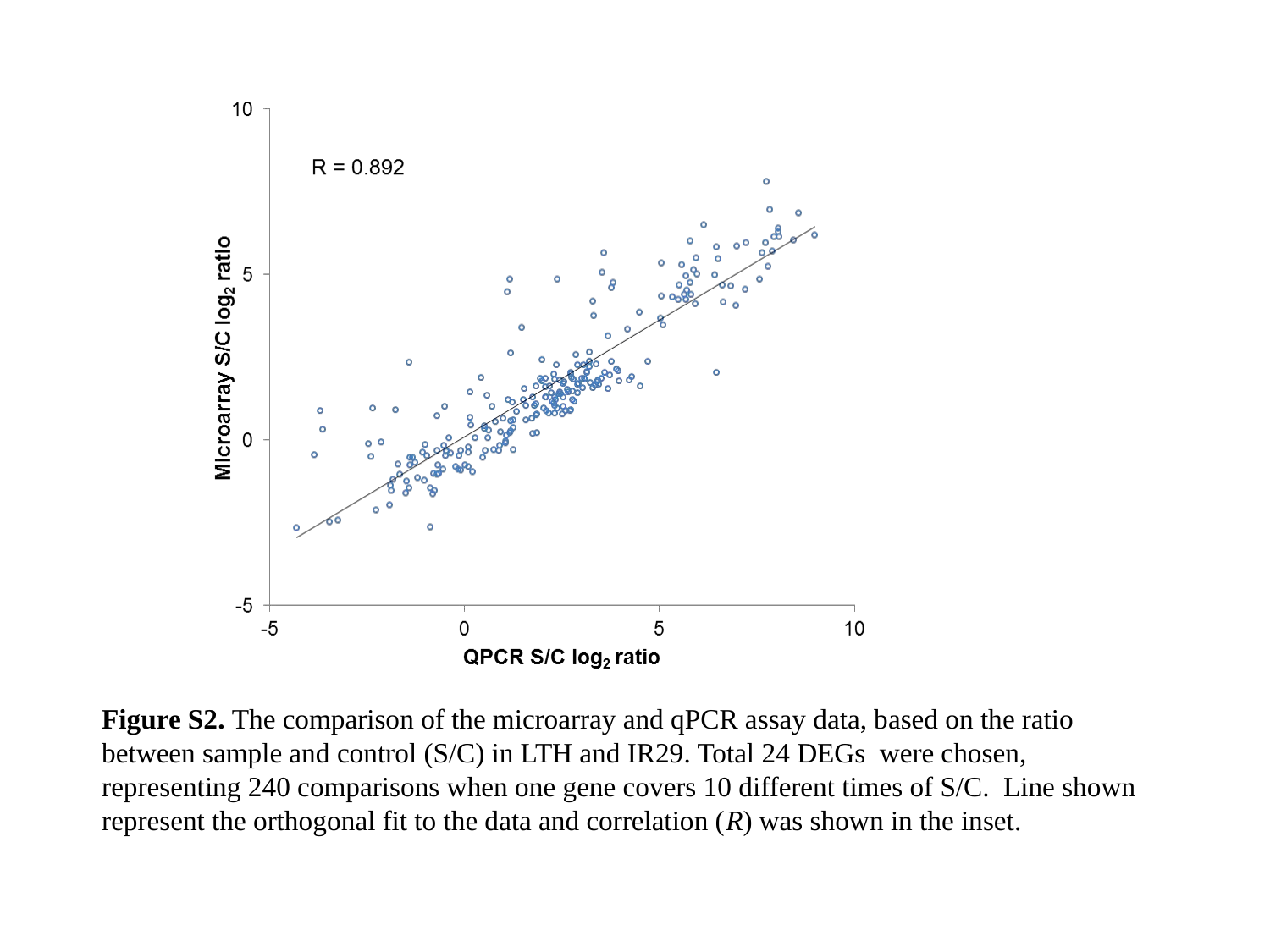

Figure S2. The comparison of the microarray and qPCR assay data, based on the ratio between sample and control (S/C) in LTH and IR29. Total 24 DEGs were chosen, representing 240 comparisons when one gene covers 10 different times of S/C.  Line shown represent the orthogonal fit to the data and correlation (R) was shown in the inset.
