# Supplementary material for: Comparative Transcriptome Profiling of Chilling Stress Responsiveness in Two Contrasting Rice Genotypes
Source: PLoS One. 2012 Aug 17;7(8):e43274. doi: 10.1371/journal.pone.0043274 (PMC3422246; doi:10.1371/journal.pone.0043274)
Supplement: Table S1 — List of gene primers used for quantitative RT-PCR. (DOC) [file pone.0043274.s003.doc]

**Table S1.** List of primers of genes for the quantitative RT-PCR

| **Gene name** | **Forward primer** | **Reverse primer** |
| --- | --- | --- |
| Os02g0618200 | 5' ACTTGAGGGTTGCTGAGTCTTC 3' | 5' CCATCATCCCTGGGTAATAAAA 3' |
| Os03g0284100 | 5' TCCCTAGTCACTCAGATCAACG 3' | 5' AGTCATCATTCTCCACCAGCA 3' |
| Os07g0695100 | 5' AAAGTGCCAGAAGGGAAAGAT 3' | 5' AGTGCTGTGCAGGATGAAATG 3' |
| Os09g0532400 | 5' CGGAGTCATCTAAGTGCTAATCG 3' | 5' ATAACCGTGGCACAGCAATCT 3' |
| Os11g0157600 | 5' AAGGTCAGGTATCATAGCAGGAA 3' | 5' GCCAATAAACAACCGACAAAA 3' |
| Os08g0157600 | 5' CCACCGTAGATTTAGGAGCAG 3' | 5' TAATAGCGGCATTCTTGTTGA 3' |
| Os06g0728700 | 5' CTTGCTCCTGGCTCATCTTCA 3' | 5' GGGACTGGGACGGATCTATCA 3' |
| Os04g0583900 | 5' ACCATAATGAGGGTGTTACTTGC 3' | 5' TCTTCGTTTCTGTTTCCGTTT 3' |
| Os02g0685200 | 5' GGACACGTCACGCCACAGTAC 3' | 5' CATCCAACATTTCCTCGTCTTTC 3' |
| Os10g0561400 | 5' GAGTCAGATACTTCTGCCGTTGC 3' | 5' CTGTGCCCTTGTTGGTGGATT 3' |
| Os01g0841500 | 5' AGGATGTGCTTGTGCCGTTAG 3' | 5' ACTGCCTTCCTCAATGTCTCG 3' |
| Os06g0127100 | 5' ACTTCGACCAGCCGTCCTACT 3' | 5' CTCCCTACCAGCTCAGCCATT 3' |
| Os09g0522200 | 5' TGCCTCAACTTCGCCGACTC 3' | 5' GCAACTTGTTCCATCACATTACCG 3' |
| Os09g0522000 | 5' CGACGACGGGTTCAGGTTCG 3' | 5' TTGCCATTCATCCTCAGTCTCATT 3' |
| Os02g0624300 | 5' GAGGAAAGGTCGGCAATGGG 3' | 5' GGTCGTGGAGGTGGATGATGG 3' |
| Os08g0473900 | 5' GTGAAGATCGGGACGAGGTAC 3' | 5' GAACGCTACAATCGGATACAAAA 3' |
| Os07g0685700 | 5' GCTTCTTTCCTCCTTTCTTCCTCC 3' | 5' CGCTGCTCCCATCATCTCCTC 3' |
| Os02g0669100 | 5' GCGACTCGTCACAGCTCAAAC 3' | 5' TCACCTCCTCTTCCTCATCACT 3' |
| Os10g0553300 | 5' GCAGAAGGGAGGAACATAACA 3' | 5' TGCCTAAGACGACAGGGACAG 3' |
| Os04g0608900 | 5' ACTTTGGAGTTGCCAGGATTG 3' | 5' ACTACTTCGGTGAATGGAGGG 3' |
| Os12g0139300 | 5' AATGCTCGGACATTTAACCCT 3' | 5' GCACAACTCTGCTGCCTACTG 3' |
| Os01g0777300 | 5' AATACCGACAGGCTCTTTCTT 3' | 5' CCAGTCTCGCAGTTATCTTCA 3' |
| Os10g0167600 | 5' AGTATTCAGCCAAGACCTTCAGA 3' | 5' GCTAATTCCCTCCTTACAACCA 3' |
| Os06g0142200 | 5' CGCACCTGAACACCTCAAGAA 3' | 5' TGGCTAATGGCAGATACGACA 3' |
