# Supplementary material for: Comparative Transcriptome Profiling of Chilling Stress Responsiveness in Two Contrasting Rice Genotypes
Source: PLoS One. 2012 Aug 17;7(8):e43274. doi: 10.1371/journal.pone.0043274 (PMC3422246; doi:10.1371/journal.pone.0043274)
Supplement: Table S4 — Summary of differentially expressed genes under chilling stress and recovery conditions. (DOC) [file pone.0043274.s006.doc]

**Table S4.** Summary of differentially expressed genes under chilling stress and recovery conditions.

| **Time Point** | **LTH-Up** | **LTH-Down** | **Subtotal** | **IR-Up** | **IR-Down** | **Subtotal** |
| --- | --- | --- | --- | --- | --- | --- |
| 2h | 1166 | 878 | 2044 | 923 | 1157 | 2080 |
| 8h | 1222 | 873 | 2095 | 1326 | 1405 | 2731 |
| 24h | 1461 | 1172 | 2633 | 1235 | 1634 | 2869 |
| 48h | 2091 | 2015 | 4106 | 1272 | 1885 | 3157 |
| 24R | 219 | 226 | 445 | 1173 | 1834 | 3017 |
| *Note: 24 R indicates 24 h recovery after 48 h chilling stress | | | | | | |
