# Supplementary material for: Comparative Transcriptome Profiling of Chilling Stress Responsiveness in Two Contrasting Rice Genotypes
Source: PLoS One. 2012 Aug 17;7(8):e43274. doi: 10.1371/journal.pone.0043274 (PMC3422246; doi:10.1371/journal.pone.0043274)
Supplement: Table S13 — Transcription factor genes differentially regulated in LTH and IR29 during chilling stress treatments. (DOC) [file pone.0043274.s015.doc]

**Table S13.** Transcription factor genes differentially regulated in LTH and IR29 during chilling stress treatments.

| **Genotype** | **LTH** | | | | | | | | **IR29** | | | | | | | |
| --- | --- | --- | --- | --- | --- | --- | --- | --- | --- | --- | --- | --- | --- | --- | --- | --- |
| **Time point** | **2h** | | **8h** | | **24h** | | **48h** | | **2h** | | **8h** | | **24h** | | **48h** | |
| **Regulation** | **Up** | **Down** | **Up** | **Down** | **Up** | **Down** | **Up** | **Down** | **Up** | **Down** | **Up** | **Down** | **Up** | **Down** | **Up** | **Down** |
| AP2/EREBP | 15 |  | 23 |  | 24 | 1 | 26 | 1 | 16 |  | 29 | 1 | 22 | 2 | 20 | 1 |
| WRKY | 16 |  | 14 |  | 17 |  | 16 |  | 12 |  | 18 |  | 13 |  | 14 |  |
| NAC | 13 |  | 14 |  | 15 | 1 | 16 | 1 | 8 | 2 | 8 | 1 | 11 | 2 | 11 | 4 |
| bHLH | 11 | 1 | 10 | 1 | 12 | 2 | 10 | 2 | 8 | 2 | 11 | 2 | 10 | 1 | 11 | 1 |
| MYB-related | 4 | 3 | 7 | 4 | 11 | 4 | 13 | 5 | 4 | 4 | 5 | 7 | 6 | 5 | 6 | 6 |
| MYB | 11 |  | 12 |  | 12 |  | 13 | 1 | 7 |  | 11 |  | 8 |  | 9 |  |
| Orphans | 6 |  | 8 |  | 7 | 1 | 9 | 1 | 4 | 3 | 3 | 3 | 5 | 2 | 5 | 4 |
| bZIP | 8 |  | 7 |  | 9 | 1 | 10 | 2 | 4 |  | 6 | 2 | 3 | 2 | 3 | 3 |
| C3H | 3 |  | 5 | 4 | 6 | 4 | 6 | 5 | 4 | 1 | 6 | 4 | 3 | 4 | 5 | 4 |
| Tify | 6 |  | 6 |  | 6 |  | 6 |  | 6 |  | 6 |  | 6 |  | 6 |  |
| HB | 4 |  | 7 |  | 5 |  | 10 |  | 2 |  | 6 | 1 | 2 | 1 | 5 | 2 |
| C2H2 | 4 | 1 | 3 | 1 | 4 | 1 | 6 | 1 | 3 | 1 | 7 |  | 4 | 1 | 4 | 2 |
| GNAT | 1 | 4 | 1 | 4 | 2 | 4 | 2 | 7 | 1 | 2 | 1 | 2 | 2 | 4 | 1 | 5 |
| GRAS | 5 |  | 6 |  | 5 |  | 10 |  | 4 |  | 2 |  | 4 |  | 3 |  |
| AUX/IAA | 2 |  | 3 |  | 3 | 1 | 4 | 1 | 1 |  | 8 | 1 | 3 | 1 | 3 | 1 |
| G2-like | 1 |  | 2 | 1 | 3 | 1 | 4 | 2 | 2 | 3 | 1 | 2 | 1 | 2 | 2 | 2 |
| HSF | 5 |  | 4 | 1 | 4 | 1 | 4 | 2 | 3 |  | 2 | 3 | 2 | 2 | 2 | 2 |
| PHD | 2 |  | 1 |  | 1 | 2 | 6 | 5 |  |  | 2 |  | 3 | 2 | 4 | 3 |
| others | 24 | 11 | 28 | 10 | 41 | 16 | 61 | 24 | 17 | 12 | 42 | 14 | 31 | 18 | 32 | 21 |
| **Total** | **141** | **20** | **161** | **26** | **187** | **40** | **232** | **60** | **106** | **30** | **174** | **43** | **139** | **49** | **146** | **61** |
