# Supplementary material for: Comparative Transcriptome Profiling of Chilling Stress Responsiveness in Two Contrasting Rice Genotypes
Source: PLoS One. 2012 Aug 17;7(8):e43274. doi: 10.1371/journal.pone.0043274 (PMC3422246; doi:10.1371/journal.pone.0043274)
Supplement: Table S14 — Cis -element analysis for the induced genes during ER chilling stress. (DOC) [file pone.0043274.s016.doc]

**Table S14.** Cis element analysis for the induced genes by early response phase of chilling stress

| **DEGs** | | **Common** | **IR29-Specific** | **LTH-Specific** | ***p* value§** | **Function** |
| --- | --- | --- | --- | --- | --- | --- |
| **No. of tested genes** | | **523** | **312** | **514** |  |  |
| CA[ACGT] [ACGT]TG | Total (%) | 93.7 | 94.2 | 90.3 | 0.022 | CBF3, ICE1 binding |
|  | Two or more copies (%) | 82.2 | 84.9 | 80.4 | 0.048 |  |
| [CT]ACT | Total (%) | 95.6 | 97.1 | 92.8 | 0.004 | mesophyll expression module |
|  | Two or more copies (%) | 94.1 | 95.8 | 91.1 | 0.005 |  |
| AAAG | Total (%) | 92.7 | 95.8 | 91.8 | 0.013 | Dof gene binding |
|  | Two or more copies (%) | 88.0 | 90.7 | 85.4 | 0.013 |  |
| GATA | Total (%) | 90.2 | 90.4 | 86.2 | 0.037 | chlorophyll a/b binding protein |
| GTAC | Total (%) | 89.3 | 89.4 | 82.5 | 0.003 | Hypoxic related gene binding |
|  | Two or more copies (%) | 71.9 | 74.4 | 66.9 | 0.012 |  |
| G[AG]WAAW^＃^ | Total (%) | 88.0 | 93.6 | 86.2 | 0.0005 | GT-1; light regulation |
|  | Two or more copies (%) | 70.2 | 79.8 | 70.8 | 0.002 |  |
| [ACG]CGCG[CGT] | Total (%) | 79.3 | 77.6 | 68.3 | 0.002 | Calmodulin binding |
|  | Two or more copies (%) | 63.9 | 60.9 | 53.1 | 0.014 |  |
| TAAAG | Total (%) | 51.8 | 58.0 | 50.0 | 0.013 | Dof gene binding |

^＃^W stand for [AT], ie A or T; **§**P value represents the significance between ER-IR-Specific and ER-LTH-Specific DEGs.
