# Supplementary material for: Comparative Transcriptome Profiling of Chilling Stress Responsiveness in Two Contrasting Rice Genotypes
Source: PLoS One. 2012 Aug 17;7(8):e43274. doi: 10.1371/journal.pone.0043274 (PMC3422246; doi:10.1371/journal.pone.0043274)
Supplement: Table S15 — Cis -element analysis for the induced genes during LR chilling stress. (DOC) [file pone.0043274.s017.doc]

**Table S15.** Cis-element analysis for the induced genes by late response phase of chilling stress

| **DEGs** | | **Common** | **IR29-Specific** | **LTH-Specific** | **p value§** | **Function** |
| --- | --- | --- | --- | --- | --- | --- |
| **No. of tested genes** | | **275** | **564** | **737** |  |  |
| [CT]ACT | Total (%) | 94.2 | 96.5 | 93.8 | 0.014 | mesophyll expression module |
|  | Two or more copies (%) | 92.7 | 95.6 | 83.3 | 7.84E-08 |  |
| GATA | Total (%) | 88.0 | 90.4 | 86.6 | 0.016 | chlorophyll a/b binding protein *^Ref4^* |
|  | Two or more copies (%) | 73.5 | 74.3 | 64.7 | 0.0021 |  |
| AAAG | Two or more copies (%) | 85.8 | 89.0 | 80.6 | 0.0007 | Dof gene binding |
| CAAT | Two or more copies (%) | 85.8 | 84.9 | 74.5 | 0.0002 | CAAT box |
| [ACGT] GATT | Two or more copies (%) | 81.8 | 85.8 | 76.3 | 0.0004 | Response regulator |
| CA[ACGT][ACGT] TG | Two or more copies (%) | 82.2 | 86.2 | 75.2 | 7.69E-05 | CBF3, ICE1 binding |
| GTAC | Total (%) | 82.9 | 88.8 | 84.8 | 0.017 | Hypoxic related gene binding |
|  | Two or more copies (%) | 64.0 | 73.2 | 59.7 | 3.97E-05 |  |
| ACGT | Total (%) | 78.5 | 82.6 | 76.9 | 0.006 | etiolation; erd |
|  | Two or more copies (%) | 57.5 | 59.8 | 48.4 | 0.0008 |  |
| GTGA | Two or more copies (%) | 73.5 | 76.2 | 69.1 | 0.013 | g10; pollen; pectate lyase |
| TGAC | Two or more copies (%) | 68.7 | 69.3 | 62.1 | 0.018 | WRKY; GA; MYB binding |
| C[ACGT] GTT[AG] | Total (%) | 62.9 | 66.3 | 61.7 | 0.044 | dehydration; water stress |
| TGTCA | Total (%) | 54.9 | 58.5 | 53.1 | 0.025 | HD; homeodomain |
| AATAAA | Total (%) | 51.3 | 52.3 | 47.1 | 0.031 | poly A signal |

**§**P value represents the significance between LR-IR-Specific and LR-LTH-Specific DEGs.
